# Establishment of a prognostic model for ovarian cancer based on mitochondrial metabolism-related genes

**DOI:** 10.3389/fonc.2023.1144430

**Published:** 2023-05-15

**Authors:** Chao Meng, Yue Sun, Guoyan Liu

**Affiliations:** ^1^ Tianjin Medical University General Hospital, Department of Gynecology and Obstetrics, Tianjin Key Laboratory of Female Reproductive Health and Eugenics, Tianjin, China; ^2^ Tianjin Medical University Cancer Institute & Hospital, National Clinical Research Center for Cancer, Tianjin’s Clinical Research Center for Cancer, Key Laboratory of Cancer Prevention and Therapy, Tianjin, China

**Keywords:** high grade serous ovarian cancer (HGSOC), mitochondrial metabolism, prognosis, tumor immune microenvironment (TME), bioinformatics

## Abstract

**Background:**

Mitochondrial metabolism and mitochondrial structure were found to be altered in high-grade serous ovarian cancer (HGSOC). The intent of this exploration was to systematically depict the relevance between mitochondrial metabolism-related genes (MMRGs) and the prognosis of HGSOC patients by bioinformatics analysis and establish a prognostic model for HGSOC.

**Methods:**

First of all, screened differentially expressed genes (DEGs) between TCGA-HGSOC and GTEx-normal by limma, with RNA-seq related HGSOC sourced from The Cancer Genome Atlas (TCGA) and the Genotype-Tissue Expression (GTEx) database. Subsequently, expressed MMRGs (DE-MMRGs) were acquired by overlapping DEGs with MMRGs, and an enrichment analysis of DE-MMRGs was performed. Kaplan-Meier (K-M) survival analysis and Cox regression analysis were conducted to validate the genes’ prognostic value, Gene Set Enrichment Analysis (GSEA) to elucidate the molecular mechanisms of the risk score, and CIBERSORT algorithm to explore the immuno landscape of HGSOC patients. Finally, a drug sensitivity analysis was made *via* the Drug Sensitivity in Cancer (GDSC) database.

**Results:**

436 HGSOC-related DE-MMRGs (222 up-regulated and 214 down-regulated) were observed to participate in multiple metabolic pathways. The study structured a MMRGs-related prognostic signature on the basis of IDO1, TNFAIP8L3, GPAT4, SLC27A1, ACSM3, ECI2, PPT2, and PMVK. Risk score was the independent prognostic element for HGSOC. Highly dangerous population was characterized by significant association with mitochondria-related biological processes, lower immune cell abundance, lower expression of immune checkpoint and antigenic molecules. Besides, 54 drugs associated with eight prognostic genes were obtained. Furthermore, copy number variation was bound up with the 8 prognostic genes in expression levels.

**Conclusion:**

We have preliminarily determined the prognostic value of MMRGs in HGSOC as well as relationship between MMRGs and the tumor immune microenvironment.

## 1 Introduction

Ovarian cancer (OvCa) is the most deadly gynecological malignant tumor in the world. It is estimated that OvCa will witness an increase of 19,880 cases in the US by 2022, accounting for approximately 1.306% of all cancers and 12,810 deaths (1). HGSOC is the most prevalent and most aggressive histotype of OvCa. The majority of patients with HGSOC are detected at advanced stages, and the five-year survival rate is only 20% to 30%. Due to the lack of effective methods, early screening is not effective and therefore, most HGSOC is detected at advanced stages, which leads to a high death rate from OvCa (2). As well as invading adjacent organs directly, OvCa can spread throughout the abdominal cavity through implantation (3), which makes optimal cytoreductive surgery difficult. Even in countries with abundant resources, for example, the United States and Canada, the 5-year survival rate of advanced-stage OvCa is merely 47%.

Mitochondria are central organelles at the crossroad of various energetic metabolisms. Glycolysis was initially considered a significant metabolic pathway in tumor metabolism reprogramming (i.e., the Warburg effect). However, increasing attention has been paid to the importance of mitochondria in oncogenesis, tumor progression, and neoplastic dissemination in recent years (4). During the metabolic transformation of mitochondria in tumor cells, mitochondria produce enough energy for the boosted metabolic demands and create the basis for the assembly of intracellular organelles, cytoskeletons, and membranes in newly formed cancer cells. The growth and spread of multiple human cancers are remarkably affected by inhibition of metabolic reprogramming (5, 6). Mitochondrial metabolism is required for tumor growth, indicating that targeting mitochondrial biosynthetic, bioenergetic, and redox functions may be effective in tumor treatment (7).

Changes in the mitochondrial genome (mtDNA) are associated with chemotherapeutic resistance and metastatic progression in some types of cancer (8). For example, Ni et al. found that platinum-sensitive recurrent HGSOC patients had more synonymous mutations whereas platinum-resistant recurrent HGSOC patients had more mtDNA somatic missense mutations based on the identification results of 569 germline mutations and 28 mtDNA somatic mutations (9). An increasing amount of data indicates that changes in microRNAs that regulate mtDNA-encoded mitochondrial proteins (mitomiRs) or nuclear-encoded mitochondrial proteins (mito-associated miRs) expression can be used as cancer biomarkers for cancer diagnosis and prognosis (8). Therefore, we propose mitochondrial metabolism as a new therapeutic strategy for OvCa.

In this study, we downloaded the mRNA expression profile and corresponding clinical data of OvCa patients from TCGA database and the sequencing data of normal ovarian tissues from GTEx database. The differentially expressed genes related to mitochondrial metabolism in OvCa were obtained by differential analysis, and the prognostic model of OvCa was constructed and verified, which provided a new idea for predicting the prognosis of OvCa.

## 2 Materials and methods

### 2.1 Data source

RNA sequencing (RNA-seq) data and clinical message for 376 HGSOC patients were attained from The Cancer Genome Atlas (TCGA) database. HGSOC’s RNA-seq data of control samples (88 healthy ovarian tissues) were downloaded from the Genotype-Tissue Expression (GTEx) database. The external validation set GSE26193 (Affymetrix Human Genome U133 Plus 2.0 Array) (10–12) contains 107 ovarian tumor samples expression profile data and survival information (survival time and survival status). Additionally, MSigDB was utilized to obtain the 1234 mitochondrial metabolism-related genes (MMRGs; **Supplementary Table 1**).

### 2.2 Differential expression analysis

Differentially expressed genes (DEGs) were identified between TCGA-HGSOC (n = 376) and GTEx-normal (n = 88) (HGSOC vs. normal) making use of the R package limma, based on |log2 fold change (FC)| > 1 and false discovery rate (FDR) < 0.05.

Moreover, overlap analysis was applied to identify elements belonging to MMRGs in the list of DEGs, which were referred to as differentially expressed MMRGs (DE-MMRGs).

### 2.3 Functional annotation of the DE-MMRGs

We used R package clusterProfiler to analyze the primary mechanisms of the obtained DE-MRRGs, which contains Gene ontology (GO) enrichment and Kyoto Encyclopedia of Genes and Genomes (KEGG). This research contrasted and categorized the DE-MRRGs to see their biological characteristics after GO enrichment analysis (13). The KEGG is a whole network that assists us to learn the functional interpretation of genes (14). GO includes biological processes (BP), the cellular component (CC), and molecular function (MF). The standard *P*< 0.05 was set up and the results were visualized using R package gplots.

### 2.4 Risk model construction, evaluation, and validation

We used 375 HGSOC samples containing complete survival information (survival time and survival status) from TCGA-HGSOC as the training set, mainly for prognostic gene screening and risk model validity assessment. Cox regression analysis associated with K-M survival analysis was adopted to identify the best prognostic genes. Briefly, the identified DE-MMRGs were brought into a univariate Cox regression analysis to filter variables to do with the HGSOC survival based on *P*< 0.05. Then, K-M was performed for the variables that met the above conditions. The specimens were divided into high- and low-expression groups by median gene expression in HGSOC patients, the divergence in overall survival (OS) between them were examined by log-rank, and variables satisfying the *P*< 0.05 were further brought into multivariate Cox analysis to output the supreme variable quantity for making prognostic signature. The research used a risk scoring system to appraise the performance of multi-gene prognostic signature. The formula was,


$$risk\;score=h0\left(t\right)\text{exp}\left(\beta_{1}\times gene_{1}+\beta_{2}\times gene_{2}+\cdots \beta_{n}\times gene_{n}\right)$$

A validity of the prognostic signature-based risk score for forecasting the prognosis of HGSOC patients was evaluated and valued in the training set and stand-alone external validation set. Risk scores were counted for every HGSOC sample in the corresponding dataset of prognostic genes by the above formula. The samples were split into high- and low risk populations in view of the mid-values of risk scores in dataset apiece. The mid-values of risk scores were computed using the MEDIAN function. K-M. ROC curves, which were constructed to appraise the accuracy and particularity of the risk score in forecasting patients’ OS at the first and third year respectively, assessed the differences in OS between the two subgroups.

### 2.5 Construction of nomogram

The 326 TCGA-HGSOC samples containing complete clinical information were selected as the basis for this part of the study. Clinical characteristics included stage, race, grade, age, and tumor residual disease. Univariate Cox analyses and multivariate Cox analyses were took advantage to test whether the risk score plays a Prognostic role independently of clinical characteristics or not. Univariate Cox with P<0.05 was subjected to further multivariate Cox. Variables with *P*< 0.05 generated by multivariate Cox analysis were regarded as independent prognostic factors for HGSOC. Nomogram was then constructed based on the identified independent factors of prognosis. The calibration curve’s 45 dashed lines represent the best predictions of the nomogram.

### 2.6 Gene set enrichment analysis of differentially expressed genes

We took advantage of GSEA to determine the denote of **DEGs** set between high and low risk scoring groups with eight-MMRGs-based signature through MSigDB c5.go.v7.4.symbols.gmt and c2.cp.kegg.v7.4.symbols.gmt. GSEA project was carried out by GSVA project. The (NES)| > 1, *P*< 0.05, q< 0.25 was considered to be statistically significant.

### 2.7 Immuno-infiltration correlation analysis

To observe the differences in immune cells in HGSOC samples from high and low risk populations, we used the CIBERSORT algorithm for reliable immune infiltration estimation based on the TCGA dataset (n = 375). Only samples with inverse convolution *P*< 0.05 of CIBERSORT were able to enter in the subsequent analysis. In this study, a total of 323 TCGA-HGSOC samples met the above criteria, 163 of which were in the highly dangerous population and 160 in the low-risk population. SIGLEC15, CD274, HAVCR2, CTLA4, PDCD1LG2, LAG3, TIGIT, and PDCD1 were selected as immune checkpoint-associated transcripts, and MICB, HLA-B, HLA-C, HLA-DRB5, HLA-DPA1, HLA-DRB1, HLA-DPB1, HLA-DQB2, HLA-DQA1, MICA, HLADQA2, HLA-DRA, HLA-DQB1, and HLA-A were selected as antigen molecule-related transcripts, and these 22 genes were extracted and expressed in high- and low risk populations. Moreover, Contacts between prognostic gene expression levels in the two groups and immune cells were estimated using Spearman method. The |cor| > 0.3, *P*< 0.05 was set as the standard of significance.

### 2.8 Drug sensitivity analysis

In order to further study the drug sensitivity of prognostic genes in HGSOC, a ridge regression model was constructed to forecast the drug IC50 based on cell line expression profiles from the Drug Sensitivity in Cancer (GDSC) database and TCGA gene expression profiles using the pRRophetic algorithm based on 375 cancer samples in the high- and low risk populations described above. The relationship between prognostic genes and drugs was detected according to the Spearman method. The |cor| > 0.3, *P*< 0.05 was set as the standard of significance.

### 2.9 CNVs analysis of gene signature

CNVs data participated in this study of the selected target genes. The study searched the copy number variation configuration file from TCGA portal. The percentage of CNV types (amplification and deletion) for each target gene was assessed. And the correlation with expression vocabulary of target genes and their CNVs was analyzed according to Spearman. The *P*< 0.05 was set as the standard of significance.

### 2.10 Statistical analysis

The study conducted the statistical analyses and the Wilcox was used to detect different levels of immune cells, immune checkpoints, and antigenic elements between high- and low risk population. *P*< 0.05 was considered statistically significant.

## 3 Results

### 3.1 Analysis of DE-MMRGs

**Figure 1:**
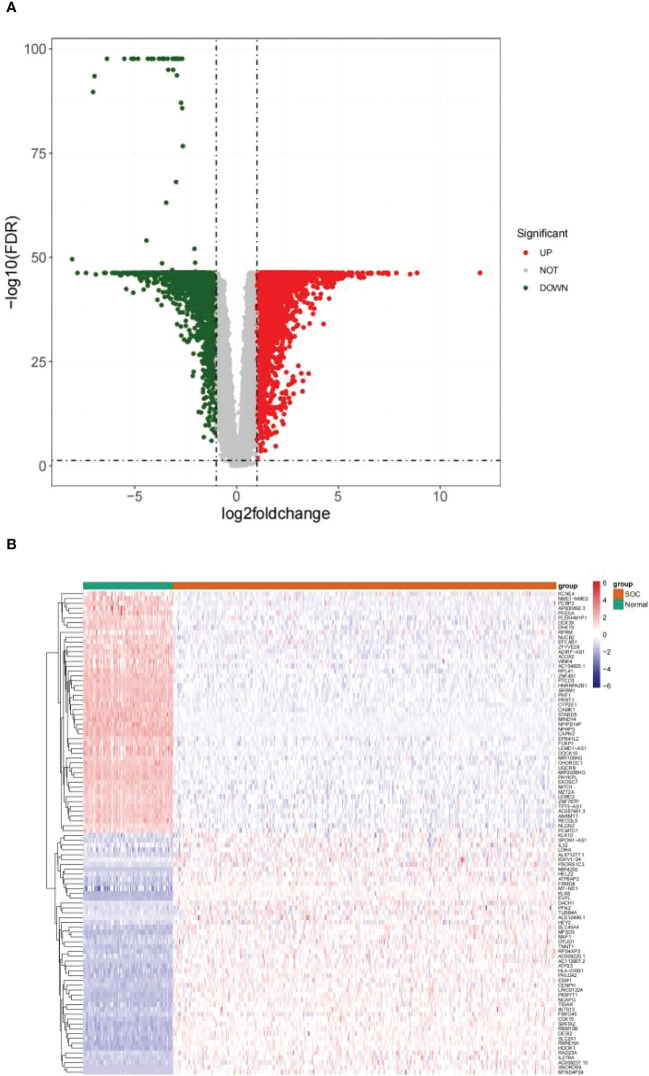
Identification of DEGs between HGSOC and normal samples. **(A)** Volcanic map of 7214 DEGs between 376 HGSOC and 88 healthy ovarian tissues. Red plots: up-regulated in HGSOC samples; green plots: down-regulated in HGSOC samples; gray plots: normally expressed mRNAs. **(B)** Heatmap of 100 DEGs (including Top 50 up- and down-regulated genes) between the two groups. Red: up-regulation; green: downregulation.

Differential analysis was performed on 376 HGSOC and 88 healthy ovarian tissues. In the aggregate, 7214 DEGs were confirmed (HGSOC vs. healthy; **Supplementary Table 2** **)**. In comparison to healthy ovarian samples, 3768 genes were up-regulated in HGSOC samples and 3446 genes were down-regulated in HGSOC samples **(** **Figure 1A** **)**. The heatmap demonstrated the expression pattern of up- and down-regulated Top 50 DEGs between the two groups **(** **Figure 1B** **)**.

**Figure 2:**
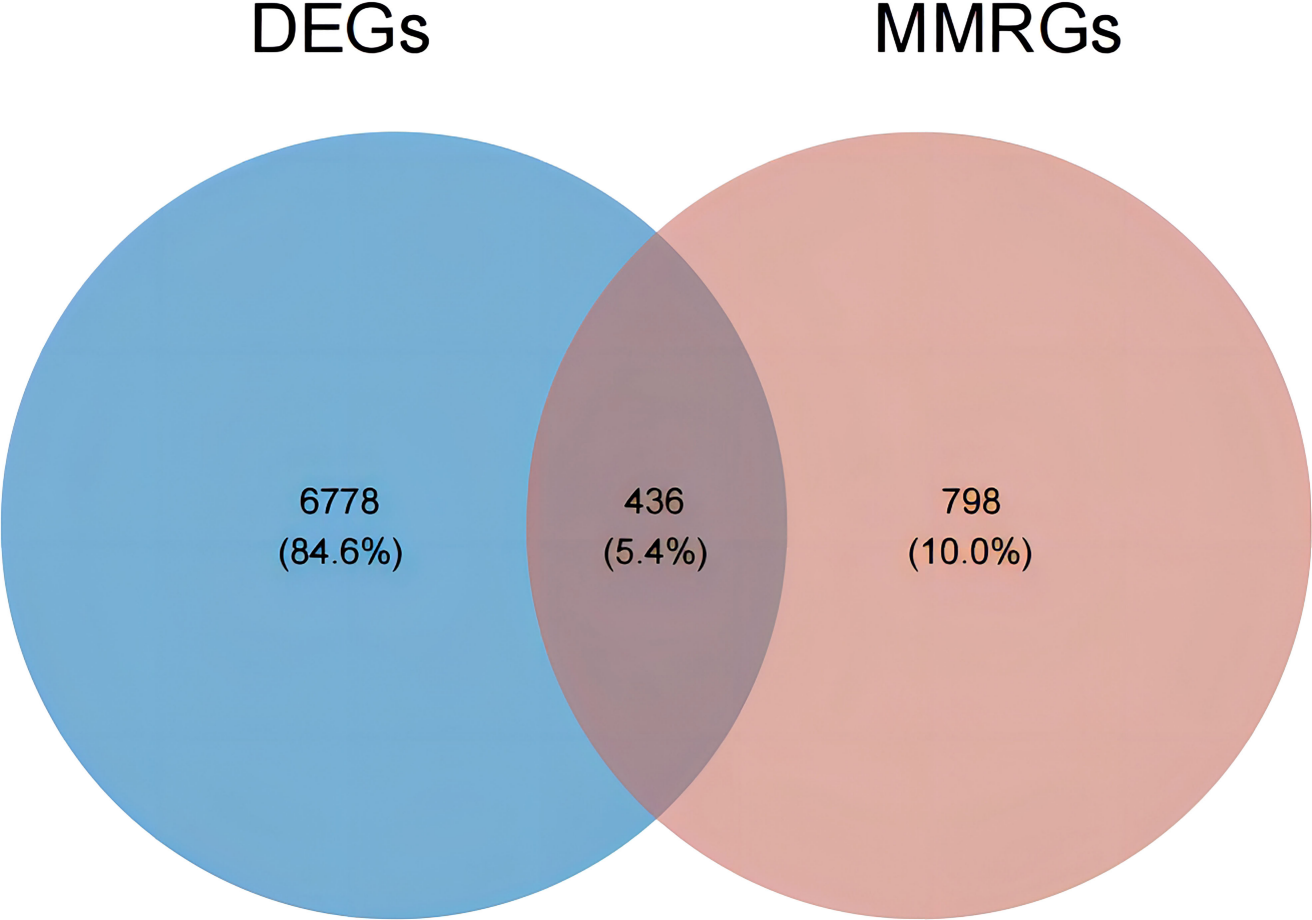
Venn diagram of DEGs and the 1234 MMRGs. Blue area: 7214 HGSOC-related DEGs; red area: 1234 MMRGs; cross area: 436 common genes.

Subsequently, an overlap analysis was performed among the HGSOC-related DEGs and the obtained 1234 MMRGs **(** **Supplementary Table 1** **)**, and the total number of 436 common genes were confirmed **(** **Figure 2** **)**. Meanwhile, 222 genes were up-regulated and 214 genes were down-regulated in HGSOC **(** **Supplementary Table 3** **)**, which were uniformly defined as HGSOC-related DE-MMRGs.

**Figure 3:**
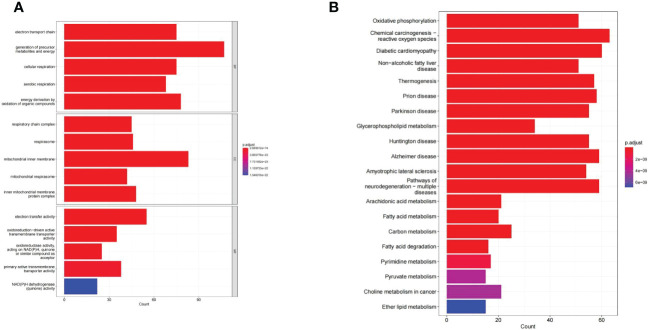
The GO and KEGG enrichment analysis of the 436 DE-MMRGs. **(A)** The top 5 terms enriched in the three categories of the GO system. **(B)** KEGG analysis enriched a total of 71 pathways. The top20 KEGG signal path was shown in the figure.

GO and KEGG enrichment analysis reveals the underlying molecular mechanisms of DE-MMRGs. The top 5 terms enriched in the three categories of the GO system were displayed in **Figure 3A**. In the GO-BP category, “electron transport chain” was the most significantly enriched term (adj. *P* = 2.69E-74, count = 75); “generation of precursor metabolites and energy” (adj. *P* = 3.33E-73) was the term involved in the most of DE-MMRGs (count = 107); also, these genes were significantly associated with “cellular respiration” (adj. *P* = 4.39E-64, count = 75), “aerobic respiration” (adj. *P* = 3.09E-61, count = 68), and “energy derivation by oxidation of organic compounds” (adj. *P* = 2.39E-56, count = 78); moreover, multiple metabolism-related processes (“fatty acid metabolic process”, etc.) were also significantly enriched. Based on the results of the CC and MF category analysis, DE-MRRGs may function as “electron transfer activity”, “primary active transmembrane transporter activity” in cellular components such as “mitochondrial respirasome”, and “inner mitochondrial membrane protein complex”. More results of GO enrichment analysis could be found in **Supplementary Table 4**. KEGG analysis enriched a total of 71 pathways **(** **Supplementary Table 5** **)**. “Oxidative phosphorylation” (adj. *P* = 2.07E-34, count = 51) was the most enriched pathway; “Chemical carcinogenesis-reactive oxygen species” (adj. *P* = 2.07E-34, count = 63) was the pathway involving the most DE-MMRGs **(** **Figure 3B** **)**. Furthermore, these genes were associated with multiple metabolic pathways, such as “Carbon metabolism”, “Fatty acid degradation”, and “Pyrimidine metabolism”.

### 3.2 Analysis of a mitochondrial metabolism-related prognostic signature

**Figure 4:**
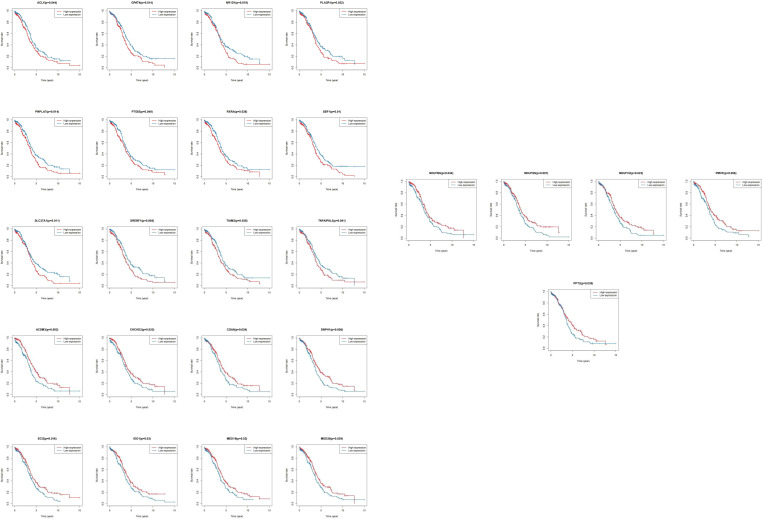
K-M survival curves between high and low expression of single gene in 25 DE-MRRGs.

We matched the transcriptomic data of 375 HGSOC samples containing survival information in the TCGA-HGSOC dataset as the training set. To explore the relationship between the 436 DE-MRRGs and the prognosis of HGSOC patients, we made a univariate Cox regression analysis of the training set. 45 of the 436 DE-MRRGs were markedly to do with the prognosis of HGSOC patients **(** **Table 1** **)**. Further, K-M curves have been performed to explore the correlation above 45 genes and the overall survival (OS) of HGSOC patients. Results indicated that the expression of 25 genes significantly differentiated the clinical outcomes of HGSOC patients **(** **Figure 4** **)**. Specifically, relatively low expression of ACLY, GPAT4, NR1D1, PLA2R1, PNPLA7, PTGIS, RXRA, SBF1, SLC27A1, SREBF1, TIAM2, and TNFAIP8L3 in HGSOC patients was conspicuously related to better OS (*P*< 0.05); whereas, high expression of ACSM3, CHCHD2, COA6, DNPH1, ECI2, IDO1, MED19, MED20, NDUFB6, NDUFS5, NDUFV2, PMVK, and PPT2 was notably linked to good prognosis in HGSOC patients. Ultimately, a prognostic signature consisting of IDO1, TNFAIP8L3, GPAT4, SLC27A1, ACSM3, ECI2, PPT2, and PMVK was constructed **(** **Table 2** **)**.

**Table 1:** 45 DE MRRGs significantly related to the prognosis of HGSOC patients.

id	HR	HR.95L	HR.95H	pvalue
TNFAIP8L3	1.577028831	1.220180577	2.038239241	0000500868
ECI2	0.731979874	0.597509048	0.896713679	0.002589998
NDUFV2	0.732921933	0.591890845	0.907556799	0.004378423
GPAT4	1.4790496	1.128214437	1.938982207	0004608334
PLA2R1	1 507083529	1.125594634	2.01786744	0.005878864
MED19	0673204675	0.50773129	0.892607061	0.005972281
SBF1	1.322092067	1.078422761	1.620818382	0.007223725
DNPH1	0.800429055	0.679416864	0.94299495	0.007772581
MED20	0 710559608	0.549478821	0.918861542	0.009184894
COA6	0 762670172	0.622029843	0.935109141	0.009185404
ACSM3	0 804624911	0.679113453	0.953332973	0.011994878
PNPLA7	1443225453	1.078429584	1 931419298	0 013592239
ACACB	1.42134615	1.074701158	1.879801527	0.013699658
NDUFB1	0.803070406	0.674411632	0.956273657	0.013821786
OSBPL5	1.356522911	1.061295217	1.733876096	0.014890436
PLGRKT	0.79437078	0.658101966	0.958855874	0 016505749
TIAM2	1.604775648	1.088009738	2.366986976	0.017062432
NR1D1	1 254156665	1.040919624	1.511076269	0.017230592
ALDH1L1	1 511132739	1.075777704	2.122671017	0.017253486
ARV1	0756724885	0.601328992	0.952278303	0.017458342
GPAT3	1 400414768	1.059745307	1.850597036	0.01788487
NDUFS5	0 801741096	0.667131437	0.963511459	0.01845595
RXRA	1.296560628	1.042822707	1.612037647	0.019422187
PTGIS	1.121033006	1.01801108	1.234480671	0.020184539
MIR210	1.235778762	1.032317775	1.479340166	0.02108282
ID01	0.910204947	0.839422586	0.986955867	0.022735806
PDK4	1 207284466	1.024672491	1.422440628	0.024370011
LTA4H	1.277063625	1.031817911	1.58060011	0.024585384
DGKD	1.394191068	1.040508403	1.868095181	0.026017471
MIGA2	1.332532009	1.031312492	1.7217299	0.028106974
PON3	0 821537668	0.689081153	0979455231	0.028424665
ACSS3	1.244013576	1.023263712	1.512386065	0.028473333
PPT2	0.744393863	0.57067223	0.970999103	0.029479794
NDUFB6	0.808208585	0.665641298	0.981310984	0.031515999
VAPA	0.697103774	0.501022307	0.969924224	0.032259677
PMVK	0788906781	0.634037525	0 981604218	0.033463157
COX8A	0.80619569	0.658819946	0.986538878	0.036479495
CSNK2B	0800333879	0.648759839	0.987321162	0.037610617
SLC27A1	1.19333812	1.008830328	1.411591058	0.039155115
PRKD1	1.277738511	1.010880692	1.615042916	0.040315362
CHCHD2	0.787843192	0.626451359	0.990814188	0.041463179
ALOX5AP	1 105273568	1.00387219	1.216917524	0.041481975
ACLY	1.270552585	1.003558586	1.608579604	0.04664848
SREBF1	1.186672143	1.002285889	1.404979148	0.04698176
NDUFA5	0.753869808	0.568967727	0.998861024	0.049079356

**Table 2:** IDO1, TNFAIP8L3, GPAT4, SLC27A1, ACSM3, ECI2, PPT2, and PMVK consist the prognostic signature.

id	coef	HR	HR.95L	HR.95H	pvalue
TNFAIP8L3	0.402461741	1.495501708	1.142916309	1.956858383	0.00334942
ECI2	-0.182903646	0.8328484	0.666118778	1.04131047	0.1085393
GPAT4	0.317123862	1.373172645	1.041450305	1.810555055	0.024585929
ACSM3	-0.174297566	0.840046891	0.704657815	1.001448878	0.051917479
ID01	-0.142984877	0.866767177	0.792024122	0.94856371	0.001885684
PPT2	-0.235623034	0.790078451	0.590589727	1.056950249	0.112529203
PMVK	-0.173628381	0.840609226	0.66476785	1.062963364	0.147051031
SLC27A1	0.193186182	1.213108632	1.005431702	1.463682266	0.043746933

### 3.3 Evaluation and confirmation of risk model

**Figure 5:**
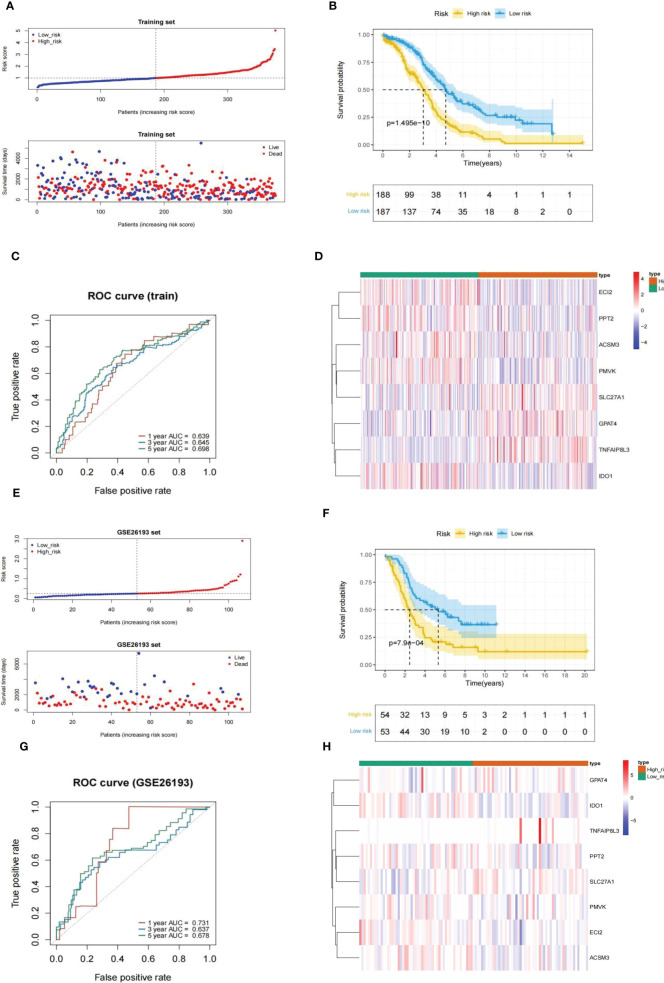
Evaluation and validation of an eight-gene-based risk model. **(A, E)** Relationship between the survival status/risk score rank and survival time (days)/risk score rank of each HGSOC sample. **(B, F) (K–M)** survival curves of the high- and low risk populations. **(C, G)** ROC curves for 1-, 3-, and 5-year overall survival. **(D, H)** The relationship between the eight prognostic genes and risk score. **(A–D)**, training set. **(D–H)**, GSE26193 set.

Every HGSOC’s risk scores of training set samples were counted in the light of previously described formula. **Figure 5A** showed that the incidence of death in HGSOC patients was climbing with an increasing risk score. The 375 HGSOC patients were then divided into high (n = 188)- and low (n = 187)-risk groups based on the midpoint of the risk score (median value = 0.993972) **(** **Supplementary Table 6** **)**,with a high-risk score indicating a poor prognosis (*P* = 1.495e-10; **Figure 5B**). Time-dependent ROC curves displayed that the risk score had AUCs of 0.639, 0.645, and 0.698 respectively in predicting OS in TCGA-HGSOC patients **(** **Figure 5C** **)**, indicating that our risk model possessed tolerable prognostic predictive performance. Moreover, the heatmap illustrated the relationship between the seven prognostic genes and risk score, with ECI2, PPT2, ACSM3, PMVK, and IDO1 negatively associated with risk score levels; while SLC27A1, GPAT4, and TNFAIP8L3 were overexpressed in the highly dangerous population **(** **Figure 5D** **)**. In agreement with the training set, we achieved comparable results in the external validation set, namely the GSE26193 dataset (**Figures 5E–H**), further evidencing the stability of the model. The expression levels of eight prognostic genes were further explored in different stage and risk groups. Except for

**Figure 6:**
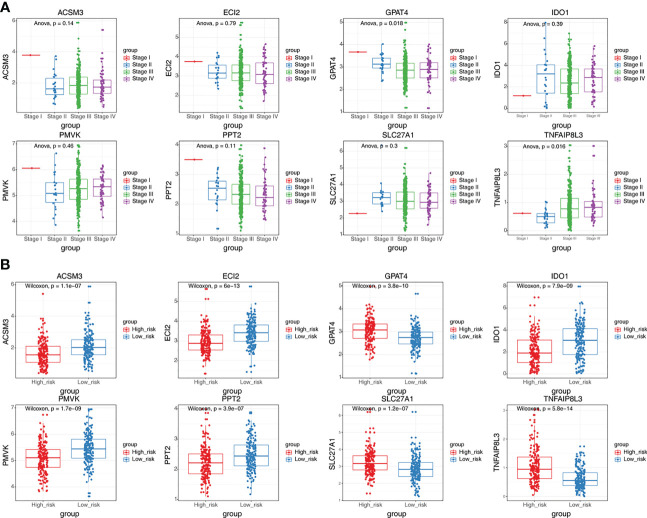
The expression differences of eight prognostic signatures in different stages and different risk groups. **(A)** The expression levels of eight prognostic signatures in patients at different stages (ANOVA). **(B)** The expression levels of eight prognostic signatures in high and low-risk groups (Wilcoxon test).

GPAT4 and TNFAIP8L3 were significantly differently expressed among stage I stage, II, stage III, and stage IV, the other 6 prognostic genes were not significantly different among different stages **(** **Figure 6A** **)**. In addition, the expression levels of prognostic signatures in the two risk groups were also analyzed, and found that GPAT4, SLC27A1, and TNFAIP8L3 were higher expressed in the high-risk group compared to low-risk group, while ACSM3, ECI2, IDO1, PMVK, and PPT2 were Lower expressed in the high-risk group than the low-risk group **(** **Figure 6B** **)**.

Next, the GSE26193 dataset (n = 107) from the GEO database was took advantage of an external independent verification set to assess the general applicability of the prognostic feature. Similarly, we worked out the exposure score for every HGSOC sample based on the equation and obtained the corresponding median value (median value = 0.2512079). All GSE26193-HGSOC specimens were categorized into high (n = 54)- and low (n = 53)-risk groups **(** **Supplementary Table 7** **)**. The risk model was also applied to the independent external validation set. Risk scores and suffer existence were presented in **Figure 5E**, with patients in the low risk population having a much longer time to subsist relative to the highly dangerous population. The small risk rating was associated with a good prognosis fpr patients analyzed by K-M survival analysis (*P* = 7.9e-04; **Figure 5F**). Then, the validity assessment analysis of the risk score in the GSE26193 dataset reported AUCs of 0.731, 0.637, and 0.678 respectively **(** **Figure 5G** **)**, indicating that the risk model enjoyed a more satisfactory predictive performance.

In conclusion, the above evidence suggested that the prognostic signature was constructed based on the eight MMRGs with a larger number of reliable predictive effectiveness and acceptable universal utilization.

### 3.4 Independent prognostic survey

**Figure 7:**
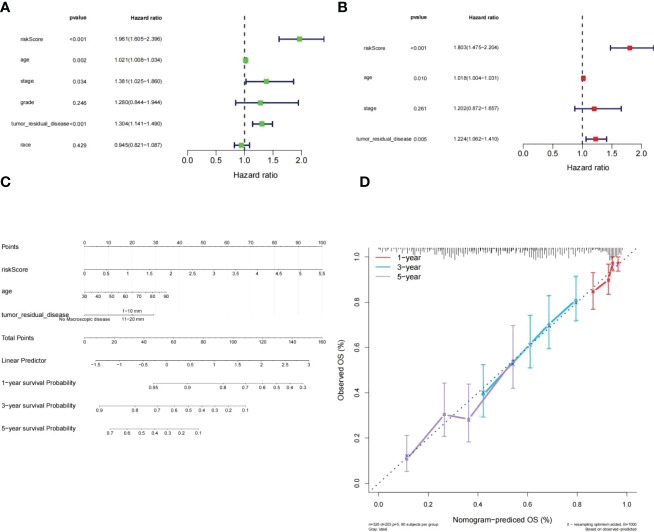
Independent prognostic investigation of the risk score. **(A, B)** Univariate **(A)** and multivariate **(B)** Cox regression analyses of risk score, age, stage, and tumor residual disease (all *P*< 0.05). **(C)** A nomogram for predicting the 1-, 3-, and 5-year OS in TCGA-HGSOC patients. **(D)** Calibration curve.of nomogram.

To assess whether or not the venture rating could predict the prognosis of patients clinical features in the clinical of HGSOC. The risk score, tumor residual disease, stage and age were meaningful relevance with prognosis in HGSOC patients (**Figure 7A**). The above 4 variables were incorporated. Ultimately, tumor residual disease, age, and risk score were considered the independent prognostic factors for HGSOC patients based on *P*< 0.05 **(** **Figure 7B** **)**.

Subsequently, we developed a nomogram model capable of predicting first-year, third-year, and fifthyear OS in TCGA-HGSOC patients **(** **Figure 7C** **)**. The calibration curve demonstrated that the survival of patients predicted by the nomogram model coincided with the actual observations, especially the OS of patients at 3 years **(** **Figure 7D** **)**.

### 3.5 Preliminary exploration of the risk score-related molecular mechanisms

**Figure 8:**
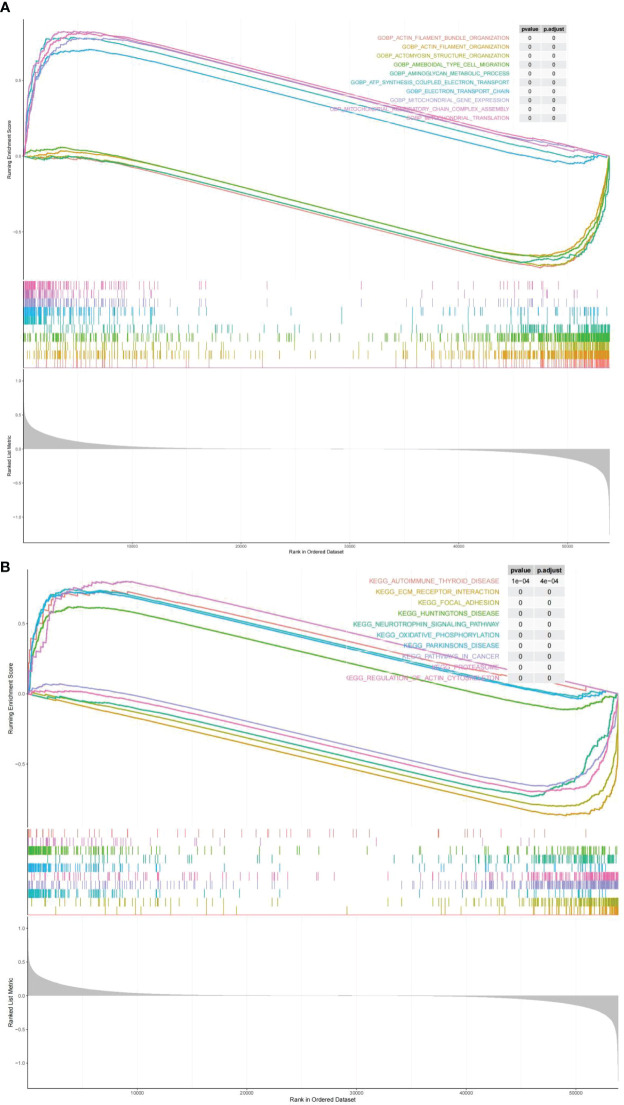
Biological processes and signaling pathways of DEGs between high and low risk groups. The results of Gene Ontology **(A)** and KEGG enrichment analysis **(B)** showed differences between high and low risk groups.

GO terms and KEGG pathways were obtained by performing GSEA on the risk score by the the R package clusterProfiler, which were differentially enriched between high and low risk. On the ground of NES values, we defined entries with NES > 1 as terms enriched, and entries of NES< -1 as terms/pathways. It indicated that “ELECTRON TRANSPORT CHAIN”, and “MITOCHONDRIAL TRANSLATION” were markedly enriched in the highly dangerous population; and “ACTOMYOSIN STRUCTURE, “AMEBOIDAL TYPE CELL MIGRATION”, as well as “AMINOGLYCAN METABOLIC PROCESS” were markedly enriched in the low risk population **(** **Figure 8A** **;** **Supplementary Table 8** **)**. KEGG enrichment analysis revealed **(** **Supplementary Table 9** **)** that the highly dangerous population was notably related to “OXIDATIVE PHOSPHORYLATION”, “PARKINSONS DISEASE PROTEASOME”, “HUNTINGTONS DISEASE”, and “AUTOIMMUNE THYROID DISEASE”; nevertheless, the low risk population was markedly correlated with “FOCAL ADHESION”, “PATHWAYS IN CANCER”, and “NEUROTROPHIN SIGNALING PATHWAY” **(** **Figure 8B** **)**.

### 3.6 Immune landscapes for HGSOC based on the risk score

**Figure 9:**
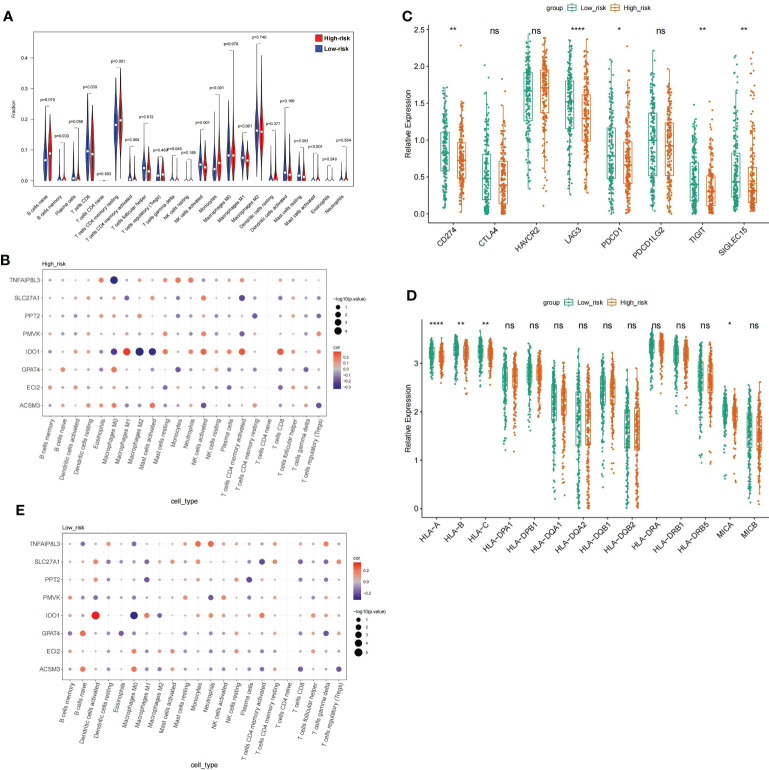
Analysis of the immune microenvironment between high- and low risk populations **(A)** V mparison of the immune cell fraction difference between the high and low-RS groups. **(D)** Expression profile of 8 genes. **(E)** The expression levels of the five immune checkpoint molecules.

GSEA illustrates that the highly dangerous population is conspicuously related to A, B, C, and D, and the low-risk population with cell adhesion processes, suggesting that the risk score probably had a bearing on the patient’s immune microenvironment. The CIBERSORT algorithm demonstrated distinctly different immune-cell inflows patterns. Highly dangerous population showed high infiltration of B cells naive, T cells CD4 memory, B cells memory period, and Monocytes; nevertheless, the low risk population showed T cells CD4 memory activated, T cells gamma delta and Mast cells activated were dramatically increased in the population of low risk **(** **Figure 9A** **)**. Subsequently, relationship between prognostic genes and 22 immunology cells **(** **Supplementary Table 10** **) (** **Supplementary Table 11** **)** was examined separately througt Spearman correlation analysis. The results revealed that in the highly dangerous population **(** **Figure 9B** **)**, IDO1 was negatively linked to Macrophages M2 (cor = -0.33041, *P* = 1.84E-05); TNFAIP8L3 expression was weakly negatively correlated with Macrophages M0 (cor = -0.30582, *P* = 7.19E-05). In the low-risk population **(** **Figure 9C** **)**, IDO1 was minimally linked to Macrophages M0 (cor = -0.31751, *P* = 4.29E-05) and proactively linked to Dendritic cells activated (cor = 0.342078, *P* = 9.54E-06). The levels of expression with five immune checkpoint molecules were distinctly different, with the levels of delivery on CD274, LAG3, PDCD1, TIGIT, and SIGLEC15 in low-risk population distinctly higher than in high-risk population **(** **Figure 9D** **)**. On the contrary, the performance rating of HLA-A, HLA-C, HLA-B, and MICA in high risk population were evidently lower than in low risk population. (**Figure 9E**).

### 3.7 Correlation of prognostic gene show and GDSC drug sensitivity in HGSOC patients

**Figure 10:**
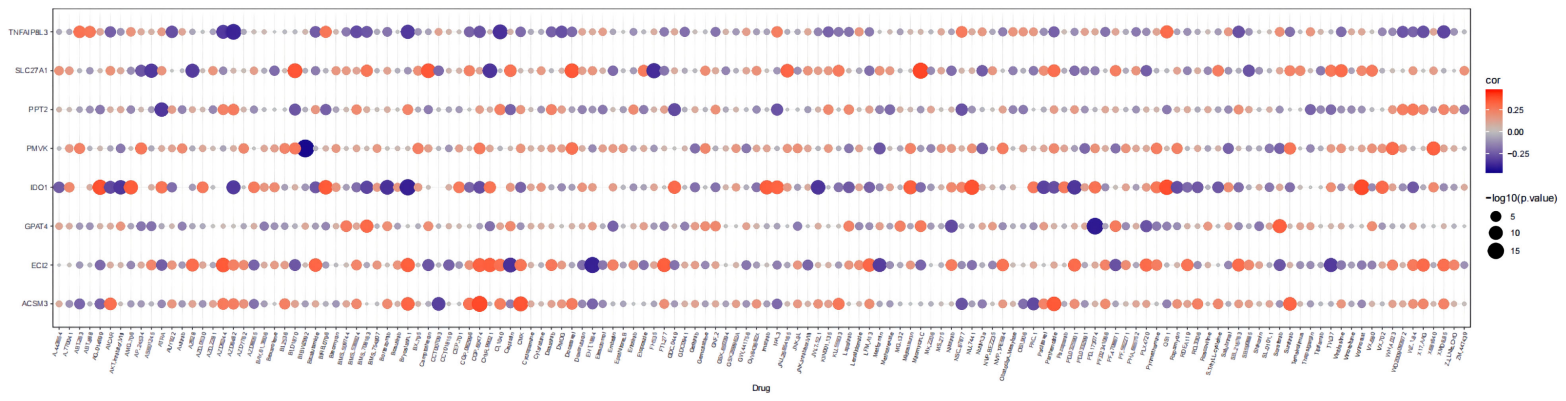
Bubble heatmap of the relationship between expression of 8 prognostic genes and drug sensitivity in HGSOC.

The links between the expression of seven prognostic genes in HGSOC and drug sensitivity were shown in **Figure 10**. The total number of the drugs obtained on the basis of the significance standard of |cor| > 0.3 and P< 0.05 is 54. Specifically, ACSM3 was minimally linked to CCT007093 (cor = -0.31769, *P* = 3.07E-10) and proactively linked to five drugs, with CGP.60474 being the strongest positive correlation (cor = 0.385081, *P* = 1.06E-14); ECI2 was correlated with 12 drugs with AZD6244 (cor = 0.346171, *P* = 5.37E-12) and EHT.1864 (cor = -0.392, *P* = 3.18E-15) being the strongest positive and negative associations; Sorafenib (cor = 0.331065, *P* = 4.83E-11) and BMS.708163 (cor = 0.304605, *P* = 1.72E-09) were positively associated with GPAT4 whereas PD.173074 (cor = -0.41038, *P* = 1.15E-16) was negatively associated with GPAT4; IDO1 was associated with up to 27 drugs, with the strongest positive association being CCT007093 (cor = 0.470611, *P*< 0.0001) and the strongest negative association with AZD6244 (cor = -0.46356, *P*< 0.05); PMVK was associated with BIBW2992 (cor = -0.44437, *P* = 1.40E-19) and X681640 (cor = 0.327402, *P* = 8.09E-11); PPT2 was only associated with ATRA (cor = - 0.34039, *P* = 1.26E-11); a total of 9 drugs were associated with SLC27A1, with the most relevant drugs being Mitomycin.C (cor = 0.390702, *P* = 4.00E-15) and FH535 (cor = -0.36538, *P* = 2.75E-13); 5 drugs (AZD6482, CI.1040, Bryostatin.1, AZD6244, and XMD8.85) were associated with TNFAIP8L3, and all showed a negative relationship with it. (cor range: -0.38199 to -0.3025, all *P*< 0.05). More particulars were available in **Supplementary Table 12**.

### 3.8 CNV analysis of prognostic genes

**Figure 11:**
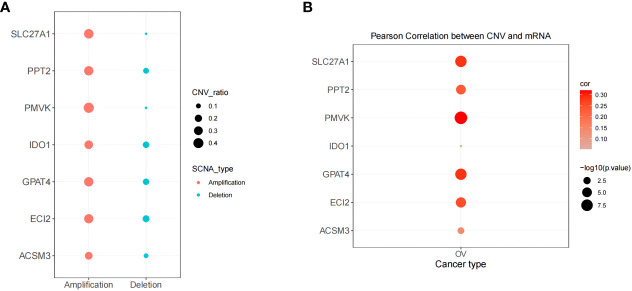
Correlation of biomarker expression levels with methylation and copy number variation levels correlation. **(A)** Copy number variation pattern of 7 gene signatures. **(B)** Pearson correlation between CNV and mRNA.

The above systematic analysis showed that aberrantly expressed prognostic genes in SOC could significantly interfere with the clinical results of HGSOC patients. Then, the study further explored the biological mechanisms of abnormal expression of seven prognostic genes (TNFAIP8L3 not matched to CNV information) from the copy number level dimension. As shown in **Figure 11A**, the 7 prognostic genes were dominated by amplified variants, with PMVK having the highest amplified variant rate of 0.418 and ECI2 having the highest deletion variant rate, but which is only 0.182. Detailed CNV rates of the 7 genes were available in **Supplementary Table 13**. Meanwhile, the study detected a notable association of copy number variation and description levels of the 7 prognostic genes **(** **Figure 11B**; **Supplementary Table 14** **)**, suggesting that genomic copy number alteration affects the expression quantity of gene in RNA-seq. Consequently, the aberrant expression of these genes in HGSOC were variant likely because of the number of copies.

## 4 Discussion

Ovarian epithelial cancer is the fifth most prevalent cause of cancer mortality in women and the main cause of gynecologic cancer deaths in the United States (15). Mitochondria plays an important role in oncogenesis, tumor progression, and tumor dissemination. Changes of mitochondrial metabolic pathways to regulate bioenergetics or anabolism contributes to the metabolic reprogramming of tumor cells (10). Inhibiting mitochondrial metabolism has become increasingly popular in the treatment of cancer. In our analytical process, the DEGs between HGSOC and GTEx-normal samples were systematically investigated. 8 MMRGs (IDO1, TNFAIP8L3, GPAT4, SLC27A1, ACSM3, ECI2, PPT2, and PMVK) associated with the prognosis of HGSOC patients were detected by Cox proportional hazards regression analysis and survival analysis. Meanwhile, by analyzing the expression changes of 8 MMRGs between high-risk and high-risk groups, we know that SLC27A1, GPAT4, and TNFAIP8L3 were highly expressed in the prognosis of high-risk patients, which may be tumor promoting factors for OvCa. ECI2, PPT2, ACSM3, PMVK, and IDO1 were low expressed in the prognosis of high-risk patients, which may be tumor suppressor factors. Subsequently, in order to validate the prognostic model, the GSE26193 dataset was used and the expression of the 8 MMRGs was evaluated using CNVs analysis. The results proved the feasibility of constructing a prognostic model with these MMRGs.

To release active immunosuppressive metabolites, indoleamine 2,3-dioxygenase (IDO1) catabolize the first step of tryptophan (Trp) catabolism along the kynurenine pathway (KP) and produce a significant effect (16). The tryptophan-kynurenine pathway and IDO1 have been recognized as pivotal mechanisms in immune escape of cancer, and inhibition of the latter might be a promising cancer treatment strategy (17). T cell proliferation is arrested with tryptophan depletion, and the general control nondepressible-2 (GCN2) kinase is activated to induces the stress response (16). Additionally, kynurenine (Kyn) encourages CD4+ T cell differentiation into immunosuppressive regulatory T (Treg) cells by activating aryl hydrocarbon receptor (AHR) (18). When CD8+ T cells are infiltrated and other immunosuppressive pathways are activated in tumors, IDO1 is expressed by stromal cells in TME and induced by IFN (19, 20). The boosted IDO1 activity has been proven to promote the development of an immunosuppressive microenvironment in cancer that inhibits antitumor immune responses (21). In this study, we found that IDO1 was associated with up to 27 drugs, with the strongest positive association with CCT007093 (cor = 0.470611, *P*< 0.0001) and the strongest negative association was with AZD6244. Therefore, inhibition of IDO1 activity may enhance the sensitivity of OvCa cells to chemotherapy agents and may serve as a potential target for anti-ovarian cancer therapy.

Enoyl-CoA (Δ) isomerase 2 (ECI2) encodes an enzyme involved in lipid metabolism, and researches indicate that a decrease in ECI2 expression results in decreased glucose utilization, fatty acid accumulation, and downregulation of cell cycle-associated genes, thus exerting a significant effect on glucose and lipid metabolism (22, 23). ECI2 probably mediates the interactions between mitochondria and peroxisomes (24). Currently, the exact role played by ECI2 in OvCa awaits further exploration.

It is known that Tumor necrosis factor-alpha-induced protein 8-like 3 (TIPE3, also called TNFAIP8L3) can strengthen the transduction of signals by phosphatidylinositol-3-kinase (PI3K). Therefore, inflammation, infection, immunity, and the occurrence and development of cancer are among the pathophysiological processes that they participate in (25–28).. Researches have discovered that the TIPE3 expression elevated in esophageal cancer (25), lung cancer (29), breast cancer (30), OvCa (31), and glioblastoma (32). The expression of TIPE3 has a positive correlation with tumor size, pathological stage, lymph node metastasis and other malignant clinicopathological characteristics (30, 32, 33). Thus, TIPE3 can serve as a new marker of intraperitoneal and lymphatic metastases (31). Additionally, the overexpression of TIPE3 in platinum-resistant EOC has been linked to dissatisfactory survival and metastasis. Compared with platinum-sensitive disease, TIPE3 may predict EOC platinum-resistance and poor outcome (28).

Recently, researches have revealed that reprogramming of lipid metabolism is crucial in tumor microenvironments, which involved regulating cancer cell malignant biological behavior (34–36). Fatty acids (FA) played an important role in hyperplastic tumors by sustaining cell renewal and mitosis (37). SLC27A1/FATP1 is an integral membrane protein that has a vital effect on lipid metabolism *via* the regulation of long-chain fatty acid uptake (38). Also, SCL27As (SCL27A1-6) significantly influences the malignant tumors progression. By downregulating SLC27A2, cisplatin resistance in lung cancer may be induced *via* the Bmi1-ABCG2 pathway, which can lead to cisplatin chemotherapy resistance in OvCa (39). However, a study of OvCa found that the decrease in FATP1 levels was related to LPL and mitochondrial β-HAD levels. The malignant metabolic alteration in cancer may result from a decrease in FATP1 expression (40). Nevertheless, we do know little about the function of SLC27A in OvCa.

By interacting with medium-chain fatty acids on the outer mitochondrial membrane, ACSM3, a subunit of CoA ligases, produces acyl-CoA (41) and plays a remarkable part in the progression of many diseases (42). In this study, we found that ACSM3 takes part in the first step of fatty acid metabolism. Compared to high expression of ACSM3, low levels of ACSM3 expression in OvCa patients may be associated with poorer overall survival. Shu et al. confirmed that ACSM3 expression was dependent on TP53 in OvCa, and there was a negative correlation between mRNA expression of ACSM3 and TP53 activation. Knockdown of ACSM3 can enhance the sensitivity of OvCa patients to paclitaxel and docetaxel (42). So ACSM3 expression can be used to predict the OvCa’s response to taxane. Another study reported that overexpression of ACSM3 suppress proliferation, migration, and invasion of OvCa cells by inhibiting integrin β1/AKT signal pathway (43). According to the results of the above-mentioned studies, combined with the results of several studies, we believe that ACSM3, as a tumor suppressor gene, may prove to be an ideal therapeutic target for treating OvCa. However, detailed molecular mechanisms underlying ovarian tumor-suppressive effects of ACSM3 need further investigation.

Immunotherapy has emerged as a promising strategy to treat various types of cancers (44). In order to regulate immune responses, PD-L1 is expressed on various immune cells, consisting of T cells, B cells, NK cells, macrophages, monocytes, and dendritic cells (45). PD-1 suppresses cytotoxic T-cells (CTLs) and stimulates regulatory T-cells (Tregs) through interactions with PD-L1, and inflammation and autoimmune diseases can be prevented by preventing excessively active immune responses (46). Anti-(programmed cell death)PD-1/PD-L1 therapy demonstrates great efficacy in combating various cancers which include but are not limited to hematological tumor, skin cancer, lung cancer, liver cancer, bladder cancer and kidney cancer, but further researches on PD-1/PD-L1 therapy in OvCa remains to be done (47)..

Interestingly, it has been shown that LAG3 and PD1, which are co-expressed by tumor antigens CD8+T cells, were damaged in the interferon -γ and tumor necrosis factor -α production, whereas the LAG3 and PD1 blocking restore the effect function of human ovarian tumor antigen T cells at higher levels than a single additive blocking LAG3 or PD1 alone. Their results reveal that the association of LAG3 with PD1 leads to their rapid trafficking to the immunological synapse, resulting in a synergistic inhibitory effect on T cell signaling (48).

Siglec15 belongs to the sialic acid-binding immunoglobulin-like lectin family (49, 50) and shares a high degree of structural homology with PD-L1 (51). There was a broad upregulation of Siglec15 in many human cancer cells and tumor-infiltrating immune cells (47, 51). Wang et al. found that Siglec15 suppresses immune function by inhibiting CD8+ T cell proliferation, and Siglec15 inhibitors are effective in reversing this suppression (49). Another study has confirmed that blocking Siglec15 by monoclonal antibody can inhibit tumor growth in mice to a certain extent (52). Therefore, further study of Siglec15-related immunotherapy and its regulatory mechanism may provide a new perspective for the treatment of OvCa.

The T-cell immunoglobulin and ITIM domain (TIGIT) is a renowned immune checkpoint molecules inhibiting T-cell functions. TIGIT is only expressed on lymphocytes and in particular on natural killer (NK) cells, effector and regulatory CD4+ T cells and CD8+ T cells (53). As a negative checkpoint on the immune response to tumors, TIGIT shares similar functions with PD-1. A study of OvCa found that TIGIT increased the CD4+ Treg response and mediated immunosuppression in the OvCa model; Therefore, blocking TIGIT played a therapeutic role in OvCa models, so there is potential therapeutic benefit from inhibiting TIGIT (54).

## 5 Conclusions

Cellular metabolic flexibility plays an important role in the effectual hyperplasia and aggressiveness of HGSOC. We constructed a MMRGs-related prognostic signature based on IDO1, TNFAIP8L3, GPAT4, SLC27A1, ACSM3, ECI2, PPT2, and PMVK, and preliminarily determined the prognostic value of MMRGs in HGSOC by bioinformatics analysis. Researching OvCa through the lens of MMRGs and the TME allows a new perspective for the study of OvCa. Additionally, according to our study, the metabolic biology of cancer cells differs from that of healthy cells, which lay a solid foundation for subsequent targeted therapies of mitochondria-related genes in OvCa. Thus, these eight genes are likely to have an effect on mitochondrial metabolism related to OvCa, but we will further investigate the role of these eight genes played in treating HGSOC.

## Data availability statement

The datasets presented in this study can be found in online repositories. The names of the repository/repositories and accession number(s) can be found in the article/**Supplementary Material**.

## Author contributions

CM: data collection, analysis and interpretation of data, paper writing YS: thesis modification. GL: experiment design, thesis modification, and fund acquisition. All authors contributed to the article and approved the submitted version.
